# Factors influencing vaccination decisions in patients with inflammatory rheumatic and musculoskeletal disease: a qualitative approach

**DOI:** 10.1186/s41927-025-00608-6

**Published:** 2026-01-07

**Authors:** Anastasia Suslow, Romy Lauer, Uta Kiltz, Chantal Giehl, Kerstin Hellwig, Theresa Oganowski, Thomas Grüter, Maria Zacharopoulou, Andreas Stallmach, Anika Franz, Ursula Marschall, Joachim Saam, Catharina Schumacher, Stephanie Stock, Dusan Simic, Arim Shukri, Kathrin Schlößler, Ina Carola Otte, Horst Christian Vollmar, the VAC-MAC consortium, Kerstin Hellwig, Theresa Oganowski, Thomas Grüter, Nina Timmesfeld, Marianne Tokic, Jale Basten, Robin Denz, Katharina Meiszl, Romy Lauer, Ingo Meyer, Katja Blaschke, Heike van de Sand, Carolin Heinen, Stephanie Stock, Dusan Simic, Arim Shukri, Uta Kiltz, Maria Zacharopoulou, Horst Christian Vollmar, Anastasia Suslow, Ina Carola Otte, Chantal Giehl, Andreas Stallmach, Anika Franz, Ursula Marschall, Joachim Saam, Catharina Schumacher, Katja Matthias, Daniel Salas, Jill-Marie Fix, Sarah Peitz, Philipp Lutz, Iris Hermanski

**Affiliations:** 1 Institute of General Practice and Family Medicine (AM RUB), Medical Faculty, Ruhr University Bochum, Universitätsstraße 150, 44801 Bochum, Germany; 2 Department of Medical Informatics, Biometry and Epidemiology, Ruhr University Bochum, Bochum, Germany; 3 Ruhr University Bochum, Bochum, Germany; 4 Department of Geriatric Medicine, Marien Hospital Herne, Ruhr University Bochum, Herne, Germany; 5 Department of Neurology, St. Josef Hospital, Ruhr University Bochum, Bochum, Germany; 6 Department of Internal Medicine IV (Gastroenterology, Hepatology and Infectious Diseases), Jena University Hospital, Jena, Germany; 7 Department Medicine and Health Services Research, BARMER Institute for Health System Research, Wuppertal, Germany; 8 Department of Health Services Research, Institute of Diversity Medicine, Ruhr University Bochum, Bochum, Germany; 9 Department of Neurology and Stroke Unit, Klinikum Lippstadt, Lippstadt, Germany; 10 Faculty of Medicine and University Hospital Cologne, Institute of Health Economics and Clinical Epidemiology (IGKE), University of Cologne, Cologne, Germany; 11 Rheumazentrum Ruhrgebiet, Herne, Germany

**Keywords:** Vaccination hesitancy, Inflammatory rheumatic and musculoskeletal disease, Vaccination barriers and facilitators, Qualitative content analysis, Physician–patient communication

## Abstract

**Introduction:**

Patients with inflammatory rheumatic and musculoskeletal diseases (iRMD) have an increased risk of infections due to immunosuppression and autoimmune disease. While vaccinations are an important preventive strategy, vaccination coverage remains insufficient in Germany. The study aimed to identify barriers and facilitators for vaccination uptake from the perspective of iRMD patients, general practitioners (GPs), and rheumatologists.

**Methods:**

We conducted semi-structured, qualitative interviews with German iRMD patients (*n* = 15), GPs (*n* = 10), and rheumatologists (*n* = 5). Data were analyzed using Kuckartz’s structured content analysis. The analysis focused on attitudes towards vaccination, information needs, decision-making, and perceived role distribution in care.

**Results:**

A trust-based doctor-patient relationship and consistent, comprehensible information promoted willingness to vaccinate. Barriers included uncertainties regarding immunosuppressants, unclear responsibilities between GPs and rheumatologists, and inconsistent or conflicting medical recommendations. Patients desired a proactive approach from physicians and clearly assigned responsibilities. Physicians emphasized interprofessional exchange but stated time and structural challenges.

**Conclusion:**

The results underline the importance of coordinated communication and clear responsibilities in the vaccination process for iRMD patients. To increase vaccination rates among patients with iRMD, the focus should be on targeted information services, improved allocation of tasks between GPs and rheumatologists, timely scheduling of vaccinations (ideally before initiating immunosuppressive therapy), clear responsibilities for initiating and coordination of vaccination, and a structured, transparent flow of evidence-based information between specialists. The results provide a basis for the development of practical intervention strategies to increase vaccination uptake in this high-risk group.

**Trial registration:**

The study was registered at the German Register of Clinical Studies (DRKS): https://drks.de/search/de/trial/DRKS00031559 (Registration Date: 28.08.2023).

**Supplementary Information:**

The online version contains supplementary material available at 10.1186/s41927-025-00608-6.

## Introduction

Patients with inflammatory rheumatic and musculoskeletal diseases (iRMD) are particularly vulnerable to infections due to both the high inflammatory burden associated with their disease and the use of immunosuppressive medications [1–4]. In particular, patients with iRMD are at elevated risk for influenza, pneumococcal disease, and other infections, especially at older ages [3, 5]. In Germany, vaccinations are mainly provided in outpatient care, particularly by general practitioners (GPs). All vaccines recommended by the Standing Committee on Vaccination (Ständige Impfkommission, STIKO) at the Robert Koch Institute, with additional guidance for patients with chronic conditions, are reimbursed by statutory health insurance (about 90% of the population) and private health insurance (about 10%), patients do not incur additional costs. Access to specialists is flexible: patients may be referred to by GPs but can also see rheumatologists, neurologists, gastroenterologists without a referral, unlike in gatekeeping systems. Consequently, coordination between primary and specialist care varies, which affects responsibilities for vaccination counselling, documentation, and implementation [2, 6].

Despite these recommendations, vaccination coverage among iRMD patients in Germany remains inadequate [3, 7–9]. According to the literature, only around 25% of iRMD patients adhere to current vaccination guidelines [7, 10].

In order to make sure that this high-risk group of patients achieves complete vaccination coverage, the vaccination status should be checked regularly [2, 5, 11–13].

While this issue is well known, the question remains why iRMD patients are often less well vaccinated than recommended.

The aim of the study was to identify barriers and facilitators for vaccination behavior from the perspective of iRMD patients, GPs, and rheumatologists. In this qualitative research, we answered the research question “What barriers and facilitating factors do patients with iRMD and their physicians experience?” In addition, we present recommended procedures to improve willingness to be vaccinated and to close the vaccination gap in iRMD patients as far as possible.

## Methods

This study is part of the project “VAC-MAC: VACcination of MS/Arthritis/Colitis patients” which investigates the autoimmune diseases Multiple Sclerosis, iRMD (e.g., Arthritis), and inflammatory bowel diseases (e.g., Colitis). The project is funded by the Innovation Fund of the Joint Federal Committee (Innovationsfonds des Gemeinsamen Bundesausschusses (G-BA)) in Germany, and the study was registered in the German Clinical Trials Register (DRKS), BfARM, Germany, DRKS-ID: DRKS00031559.

We aimed to conduct interviews with thirty participants: fifteen iRMD patients, ten GPs, and five rheumatologists. Our goal was to include fifteen patients and fifteen physicians in total [14, 15]. We assumed that we would reach thematic saturation within rheumatologists faster than with GPs, as GPs experience might represent a broader spectrum while rheumatologists could make more concise statements based on their experience. Physicians involved in the VAC-MAC projects recruited iRMD patients, GPs, and rheumatologists by using personal outreach and informational brochures, as well as snowball sampling. A purposive sampling approach was employed, with the aim of recruiting stakeholders (physicians and patients) with self-reported experience regarding iRMD or vaccination, respectively [16].

Participants could contact us via phone or email to receive detailed initial information about the study, including a consent form, data protection guidelines, and a questionnaire for collecting demographic data. Upon request, a preliminary phone call was offered to clarify questions and to get to know the interviewers. Prior to the interviews, patients were always informed in writing and orally that they could discontinue the interview at any time if they wished to do so. Our physicians who were involved in the project were available for debriefing, although this option was not utilized. All participants provided written informed consent and received compensation for their participation.

Semi-structured interviews with a high narrative component were conducted by AS and RL (Professional background: Sociologist, M.A. and Health Economist) using an interview guideline, developed by the qualitative research team, informed by a literature review, and developed collaboratively [17, 18]. We made sure to select a strong opening question that provided a compelling narrative stimulus.

The semi-structured interviews for all participants covered topics such as vaccination recommendations and implementation, the decision-making process, and their expectations for the future. The semi-structured interview guide was informed by the COM-B model and selected domains of the Theoretical Domains Framework (TDF) to ensure systematic coverage of individual determinants of vaccination behavior (e.g. knowledge and skills → Capability; communication and workflow conditions → Opportunity; decision-making and attitudes → Motivation). As organizational and contextual factors were also relevant, elements of the Consolidated Framework for Implementation Research (CFIR), particularly “Inner Setting” and “Networks & Communication”, were incorporated when constructing questions on practice routines, interprofessional coordination, and available resources [19]. The interview guidelines for patients and physicians were developed in German for this study, translated into English for publication, and can be found in the Appendix.

Depending on the participants’ preferences, the interviews were conducted via telephone, video call, or in person, either at the participants’ homes or on university premises. All Interviews took place in settings ensuring privacy and allowed for open, trustful communication; duration was sufficient to achieve in-depth, rich data. No substantial differences in content or thematic depth were observed between the different interview modes. Interviews were planned to last about one hour. The face-to-face and telephone interviews were recorded using external audio devices, while video call interviews were recorded using the platform’s built-in recording tool. The recorded interviews were then transcribed and pseudonymized by a transcription service. All quotes were translated from German into English and back into German for verification by the research team.

The interviews were analyzed according to Kuckartz’s Qualitative Content Analysis [20]. Initially, a deductive coding system, based on the question structure of the interview, was applied in MAXQDA 2024 by AS to coding the first three interviews [21]. As the coding process progressed, additional inductive codes were introduced to capture the depth and complexity of the collected data more accurately [21]. In addition, coding decisions and emerging themes were discussed in biweekly multidisciplinary team meetings (peer debriefing). To enhance the trustworthiness of the findings, a form of member check was conducted: selected interview quotes and interim results were presented in webinars to interested patients, who were invited to ask questions and provide feedback on the preliminary interpretations [22]. The translated coding trees are provided in the Appendix. The reporting of our results was oriented towards COREQ.

The demographic questionnaire for patients collected information on age, gender, disease, disease duration, and self-reported information about vaccinations received in the past ten years [6]. Participants were advised to take the information from their personal vaccination record. Physicians were asked about their age, gender, medical specialty, and years of professional experience.

## Results

We reached our target sample size of 30 participants, anticipating greater thematic diversity among GPs and more expertise-focused contributions from rheumatologists [15]. This also allowed us to achieve thematic saturation, which we have determined based on the fact that no new information could be generated from the interviews. Saturation was reached after interview 6 for GPs and interview 4 for rheumatologists, with no new codes emerging, meaning saturation was confirmed through iterative theme summaries [23]. Therefore, we concluded the recruitment process [17, 24]. Demographic characteristics of the participants are shown in Table 1. In total, we interviewed thirty people (fifteen iRMD patients, ten GPs, and five rheumatologists) between March and December 2023.

**Table 1:** Demographic characteristics of interview participants

Characteristics	
Gender (female), patients ***n (%)***	10/15 (66)
Gender (female), GPs ***n (%)***	4/10 (40)
Gender (female), rheumatologists ***n (%)***	4/5 (80)
Age of patients ***mean/median (min-max)***	55/55 (27–80)
Age of patients at disease diagnosis ***mean/median (min-max)***	43/43 (20–78)
Duration of iRMD ***mean/median (min-max)***	12/7 (1–41)
Interview duration, patients in minutes ***mean (min-max)***	22* (12–44)
Interview duration, GPs in minutes ***mean (min-max)***	23 (12–43)
Interview duration, rheumatologists in minutes ***mean (min-max)***	31 (13–54)

*Even though the average interview duration appears short, all questions were properly addressed and narrative episodes revealed information-rich answers in the short interviews. For the most part, these were interviews in which the participants answered many questions independently in a narrative flow

**Fig. 1:**
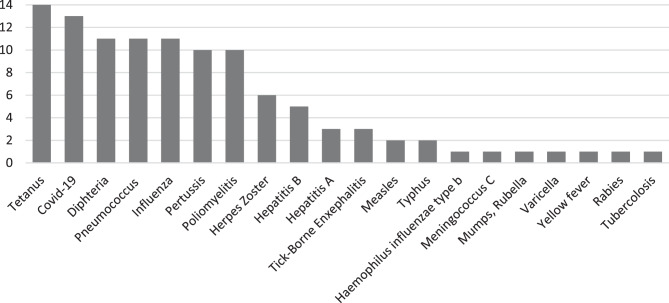
Vaccination status represented by patients in the past 10 years in iRMD patients (*n* = 15) sorted by frequency

The most frequently administered vaccinations among the interviewed iRMD patients over the past ten years included tetanus, SARS-CoV-2, pneumococcal, and influenza vaccines. Figure 1 illustrates the types and frequencies of vaccinations received by the fifteen iRMD patients. All 15 patients completed the questionnaire and provided information for all listed vaccines; no missing records were identified. Frequencies represent the number of patients who reported receiving the respective vaccine.

**Fig. 2:**
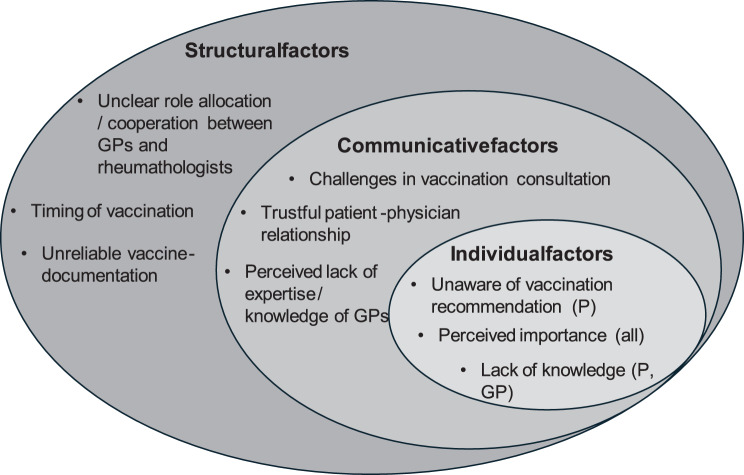
Key themes regarding low vaccination rates from the interviews. (GP =  General Practitioner; *p* = patient; all = all participants)

In the following section, we present the interview findings supported by original quotes. Our analysis of the semi-structured interviews revealed four key themes related to the potential causes of low vaccination rates among people with iRMD: a) Perceived importance of vaccinations for immunosuppressed patients, b) The challenges of vaccination consultation, c) Cooperation between physicians in the vaccination process and d) Need for improvement in vaccination-documentation. We categorized themes and subthemes to structural, communicative and individual factors as shown in Fig. 2 [25].

### Perceived importance of vaccinations for immunosuppressed patients

The interviews showed that it was extremely important to the rheumatologists to have the patients vaccinated before starting treatment or to refresh the vaccinations according to the recommendations. Especially as infections could trigger a flare, a vaccination is seen as the “lesser evil” in this case:

Rheumatologist-04: *„So ideally, if a patient comes in for an initial diagnosis and they have an inflammatory rheumatologic disease, we recommend that they update their vaccinations before starting treatment […].”*

Rheumatologist-01: *„And we know that certain infections can also trigger the disease again. […] Then sometimes a vaccination is simply the smaller pill to swallow.”*

GPs made fewer comments on this theme but overall supported the view of rheumatologists.

This approach is also preferred by patients, especially if they have already experienced infections due to immunosuppression and would therefore rather accept potential side effects of the vaccination than contract severe disease:

Patient-06: *“I have already picked up other illnesses with my immunosuppression. That’s why I know that it’s not always fun, not as harmless as it might be for other people. And that’s why you’re really protected […].”*

Patient-09: *„But when I get sick, I feel even worse, right? If I get severe pneumonia and have a high temperature, that’s worse than if I have the symptoms of a cold, isn’t it?”*

### The challenges of vaccination consultations

One challenge in vaccination consultations is that physicians often do not have sufficient time to provide their patients with detailed information:

Rheumatologist-01: *„What I see as the biggest challenge is that it simply requires information [for the patient]. And information means talking and having time. […] And neither I nor the GP have time for this, because we don’t get paid for it. If I were adequately remunerated for doing proper vaccination information here, then people would be happy to do it. […] So speaking medicine is just not worth anything.“*

Another rheumatologist, on the other hand, states that she/he has a lot of time per patient available in her or his practice, so that she or he can address patients’ questions and needs in more detail and accordingly provide more detailed advice. Experience has shown that patients are more open to receiving a vaccination if the necessity of it and the detailed procedures are discussed:

Rheumatologist-02: *„Well, it is explained at the start of treatment that the principle of the medication we give is to regulate the immune system down a little and try to calm it down. So that it doesn’t turn against itself. And so that the sensitivity to diseases or the risk is a little higher. And at that point at the latest, I would say that it makes sense to pay particular attention to protecting against the disease. I believe that patients are very, very dependent on how you phrase it. […] How seriously you say it and express your own concern for the patient.“*

One patient expressed a desire for oral information. Although information material was provided by her or his rheumatologist, she or he was hesitant regarding vaccination. From her or his point of view, the physician, no matter if specialist or GP, should take responsibility for addressing vaccination again at the next appointment:

Patient-12: *„That this is definitely better explained or given to you. And then maybe ask again, not just give them a piece of paper, but ask them again next time. Because it’s often the case that you get something in your hand and then it goes into the cupboard and that’s it, right?“*

One rheumatologist stated that she or he provides written information to the patients’ GPs and asks the patients to talk to their GP about it:

Rheumatologist-02: *„And we have an information sheet, relatively detailed, two-sided. We give it to the GP with our recommendations on it. And then we ask patients to discuss this with their GP and complete their vaccination program. […] I think it makes sense for such standardized schedules to be made available in a language and form that patients can understand.”*

This also seems to correspond to the patients’ preferences. Although it was stated above by patient-12 that she or he did not like the fact that only written information was provided, this patient believes that it makes sense to give her or him the opportunity to obtain information for themselves:

Patient-06: *„Right at the beginning, I was given a sheet of paper with the recommended vaccinations in my case. So, of course I was able to do some research first and find out from my GP what vaccinations I might be missing, which ones I might have, even if I already had them.”*

### Cooperation between physicians in the vaccination process

The patients interviewed raised the uncertainty as to which of the physicians is responsible for providing vaccination advice and, above all, for administering the vaccination itself. While the interviewed GPs considered themselves responsible for vaccination, they expressed possible gaps in their knowledge regarding immunocompetence, particularly in relation to disease-modifying anti-rheumatic drugs (DMARDs). Contrary, rheumatologists said that they are responsible for this, as they have disease-specific expertise and cooperation with the GPs has not been sufficient so far:

Rheumatologist-01: *„But that vaccination is totally important for our rheumatologic patients and that we are also the ones who have the expertise to provide the right information. Because I don’t expect a GP to have an overview and understanding of all this. They shouldn’t. That’s what I’m a rheumatologist for.“*

Rheumatologist-04: *„So initially I always thought it would be the GPs. But as this has never worked out so well in the past, the rheumatologists, i.e. internal rheumatologists, have decided that we should also take this into our own hands. Because we simply lose far too much time if we send people back [to the GP] to get the vaccinations.”*

One GP also criticized the dissatisfactory cooperation with the rheumatologists:

GP-09: *„Because then I don’t realize that an immunosuppressive therapy has to be done, and then I’m rather sad or angry when I find out, okay, he’s now been given MTX, […]. And I didn’t have the opportunity to do the whole thing beforehand. That’s annoying, of course.“*

However, the patients were not very clear about which physician they wanted to receive the information and vaccination from. On the one hand, the GP is familiar with the patient’s medical history but may lack disease-specific knowledge; on the other hand, the rheumatologist has the disease-specific knowledge but may not know the patient quite well:

Patient-06: *„And I believe that the GP is actually simply the better person to talk to. Especially because he knows you very well. No, so my GP, he only needs to look at me, he actually knows what’s wrong with me, right? And that’s not necessarily the case with many specialists, is it? They see me every six months or every three months […].”*

Patient-15: *„And I don’t think my GP has a complete overview of this because he is not the specialist for this condition. And if I have any other questions about the disease, I go to a specialist and not to my GP.”*

### Need for improvement in vaccine-documentation

In Germany, a paper booklet is currently used to document vaccinations. Thus, vaccinations are not reliably recorded:

Patient-04: *„The current vaccination card I have is, I think, my third. Because somehow it always gets lost, doesn’t it? So it would make sense, all sorts of things are registered somewhere, but why isn’t it just registered somewhere on a chip card or something?”*

A digitized vaccination card could help to keep track of vaccinations:

GP-07: *„So, what I would really like to have is a digital vaccination card. We keep one for our patients ourselves. But to be honest, we don’t always get around to entering vaccinations that have already been given into this card. […] So that would be my greatest wish. Simply digitized.“*

## Discussion

The qualitative interviews show that both patients with iRMD and their GPs and rheumatologists generally acknowledge the importance of vaccinations. At the same time, there are considerable challenges in vaccination consultations, particularly due to limited time resources and a lack of reimbursement. In addition, unclear responsibilities between GPs and rheumatologists in the vaccination process led to uncertainty among patients.

Other studies have reported that patients fear vaccinations could trigger a disease flare or may be ineffective due to immunosuppression [11, 26, 27], but these concerns were not raised in the interviews we conducted. This may be due to our study’s focus on iRMD patients, the phrasing of our interview questions, or the participants’ prior experiences with immunosuppression. It is also possible that our recruitment strategy led to selection of patients who were already relatively confident about vaccination, highlighting the importance of considering sample characteristics and study design when comparing qualitative findings across studies. Also, we did not explicitly address this issue in order to avoid steering the conversation in that direction, and none of the iRMD patients brought it up on their own. In contrast, such concerns were spontaneously mentioned by patients in our interviews with individuals diagnosed with multiple sclerosis [28]. Nonetheless, such concerns seem to persist, including the belief that vaccinations may not work at all in people with autoimmune diseases. As a result, both patients and physicians may refrain from immunization [29].

### Perceived importance of vaccinations for immunosuppressed patients

The importance of comprehensive care for iRMD patients, including consultation and, if necessary, vaccination, has also been emphasized in other studies [26]. However, some patients report that their physicians did not address the potential increased infection risk or the need for vaccination [7]. This information appears to be particularly important, as many patients are not vaccinated simply because they are unaware of the need [10, 27].

### The challenges of vaccination consultations

Our findings, along with previous research, suggest that most patients would be willing to be vaccinated if their treating physician—whether GP or rheumatologist—recommended it [30]. iRMD patients stated that they were afraid of vaccination adverse events, especially disease flares, and distrust in vaccines, especially regarding SARS-CoV-2 vaccinations [7, 11, 30]. In addition, patients should receive guidance on when treatment should be initiated and at which intervals vaccinations can be administered. Moreover, financial incentives for physicians have been shown to be effective in increasing vaccination uptake; however, such measures should be carefully considered in the context of iRMD patients, as they may not address underlying fears or mistrust [31].

### Cooperation between physicians in the vaccinations process

Our results reflect that responsibilities for recommending and administering vaccinations are not clearly defined, and existing literature indicates that rheumatologists may view GPs as responsible and vice versa. On the one hand, physicians might not be sufficiently familiar with information on (contra-)indications for vaccinations in iRMD, including administration of vaccines at an optimal timepoint in patients with immunosuppressive medication [2, 9]. Vaccinations in Germany are mainly carried out in GPs practices, and specialists tend to be reluctant to vaccinate [7, 29]. In Germany, patients can consult specialists directly, as the healthcare system does not follow a strict primary care gatekeeping model. While vaccinations (except for travel vaccinations) are free for patients, GPs receive extrabudgetary reimbursement, creating an incentive to provide them despite time constraints. Long waiting times and variable access to GPs may further contribute to uncertainty about responsibility between physicians, highlighting the need for clear role definitions and improved interprofessional coordination [32, 33]. Physicians should also be given clear guidance on which vaccinations are recommended for immunosuppressed patients and how they can best alleviate their patients’ potential concerns [9, 10].

### Need for improvement in vaccine documentation

The expressed desire by both GPs and patients for a digital vaccination record highlights a concrete and feasible opportunity to improve vaccination management. The current use of paper-based vaccination records in Germany leads to practical challenges such as loss, fragmentation, and incomplete documentation [29]. Digital solutions could improve the continuity and accessibility of vaccination records across different healthcare providers, reduce administrative burden and improve patient engagement. The introduction of a secure and widely accepted digital vaccination card could therefore be a crucial step towards improving vaccination procedures and vaccination rates among patients with chronic diseases. The introduction of electronic patient records (ePA) in Germany in 2025 has already paved the way for a digital vaccination card. Developers are currently working on specific implementation. However, the ePA is intended to give physicians insight into their patients’ treatment history.

### Practice implication

Information materials can help to address the concerns mentioned above. The main consideration is immunosuppression, which may affect vaccine efficacy. However, treatment should be prioritized and not interrupted for the sake of vaccination [10, 13, 34, 35].

In order to address all the topics mentioned in the interviews, the research team created a homepage with the necessary information requested by physicians and patients. The website http://www.vac-mac.de (last access 31.10.2025), provides all relevant information on vaccinations for iRMD patients in plain (German) language, targeting both physicians and patients. The site also offers a downloadable letter for attending physicians, brochures, and informational videos.

## Strengths and limitations

This study offers a comprehensive insight into factors that promote and inhibit vaccination from the perspective of patients with iRMD, as well as GPs and rheumatologists. The different perspectives enable a differentiated understanding of structural, communicative, and individual influencing factors. A broad spectrum of experiences was covered through the purposive selection of interview partners from different areas of care.

The patients we interviewed were well immunized according to their self-report. Thus, it cannot be ruled out that patients who were willing to be vaccinated were more willing to participate in the study, which may have introduced a selection bias. Nevertheless, our interviews were rich regarding barriers and facilitators to vaccination. However, one common barrier, the fear of worsening of the disease after vaccination, was not represented in our data. It remains unclear, whether this relies on our patient sampling or the German setting, as the theme was neither mentioned by patients nor physicians. Even though the sample size was relatively small, we were able to identify a thematic saturation in the interviews. Of course, the transferability to healthcare systems outside Germany is limited if only people in Germany were interviewed. We would like to present the views of our physicians and patients here and offer regional solutions that could potentially be expanded in the future by translating the materials we have created.

The qualitative methodology does not allow any quantitative or representative statements to be made about the frequency of certain views, but it does provide valuable hypotheses for further research and practical interventions.

## Reflexivity

The interviews were conducted by AS (Sociologist, M.A.) and RL (Health Economist). Their professional backgrounds may have influenced the way questions were asked and answers were interpreted. To minimize potential bias, the interview guidelines were developed collaboratively within the research team and applied consistently across all interviews. All transcripts were analyzed by AS, and preliminary findings were subsequently discussed with patients in webinars as a form of member check to reinforce the credibility and neutrality of interpretations.

## Conclusion

The results of this study illustrate that vaccination decisions in iRMD patients are strongly influenced by the combination of individual attitudes, medical communication, and structural conditions. The central role of GPs and rheumatologists, who can contribute to promoting the willingness to vaccinate through clear recommendations and comprehensible information, became particularly clear. At the same time, the identified barriers show that uncertainties in communication and a lack of clearly defined responsibilities negatively affect vaccination attitudes. Our findings offer practical starting points for the development of targeted interventions to promote vaccination in the context of rheumatology care.

## Electronic supplementary material

Below is the link to the electronic supplementary material.

Supplementary Material 1: Translated coding tree patients

Supplementary Material 2: Translated coding tree physicians

Supplementary Material 3: Translated guideline patients

Supplementary Material 4: Translated guideline physicians

## Data Availability

The code trees and interview guidelines are included in the Appendix. Quotes are listed in the manuscript. For data protection reasons, the pseudonymization table is not publicly available. The pseudonymized interview transcripts or parts thereof and MAXQDA files can be shared with researchers who submit a justified request stating the intended use and committing to maintain confidentiality.

## Declarations

### Key indexing terms

Rheumatoid Arthritis; Musculoskeletal Diseases; Vaccination; Immunization; Qualitative Research; Vaccination Hesitancies
