# Supplementary material for: Factors influencing vaccination decisions in patients with inflammatory rheumatic and musculoskeletal disease: a qualitative approach
Source: BMC Rheumatol. 2026 Jan 7;10:11. doi: 10.1186/s41927-025-00608-6 (PMC12849479; doi:10.1186/s41927-025-00608-6)
Supplement: Supplementary file 1 — Supplementary Material 1: Translated coding tree patients [file 41927_2025_608_MOESM1_ESM.docx]

List of Codes

| **List of Codes** | **Frequency** |
| --- | --- |
| Codesystem | 1867 |
| Responsible medical specialists | 60 |
| Exchange between the specialists | 11 |
| Frequency of appointments | 44 |
| Treatment type | 45 |
| Special consultation hours / training | 16 |
| Paternalism by physicians | 25 |
| Responsibility for vaccinations | 10 |
| Checking the vaccination status | 64 |
| Personal initiative | 52 |
| Advice on vaccinations | 122 |
| Received information material | 35 |
| Carrying out vaccinations | 71 |
| “No thought given” / ignorance | 23 |
| Missing information | 7 |
| Trust in the physicians | 37 |
| Decision-making process for vaccination | 19 |
| Protecting family members | 4 |
| Professional requirement | 15 |
| Vaccation | 4 |
| Personal evaluation | 19 |
| Vaccination-critical | 1 |
| Fundamental support for vaccination | 36 |
| “It's always been done this way” | 8 |
| Exchange with social environment | 75 |
| Reaction from the social environment | 25 |
| Disease as a parameter | 44 |
| Fear of infection with pre-existing illnesses | 18 |
| Treatment as a parameter | 87 |
| Own research | 27 |
| TV | 9 |
| Magazines / Newspaper / Articles / Lectures | 10 |
| Internet / Forums / Blog posts | 50 |
| Internal access to information (work / friends in the healthcare sector) | 15 |
| Decision-making before diagnosis | 20 |
| Worries / concerns about vaccinations | 46 |
| Exchange with physicians about worries / concerns | 33 |
| How the physicians deal with the worries / concerns | 16 |
| Side effects / after-effects / decision on vaccinations | 1 |
| Shingles | 24 |
| HPV | 5 |
| Pneumococcus | 17 |
| Measles / mumps / rubella | 10 |
| Covid-19 | 88 |
| TBE | 15 |
| Hepatitis (A / B / C) | 10 |
| Influenza | 38 |
| Meningococcus (A / B) | 6 |
| Tetanus | 22 |
| Other vaccinations | 12 |
| Reflection on the vaccinations | 0 |
| Desire for digitization | 2 |
| Desire for initiative on the part of physicians | 8 |
| Advantages of vaccination | 31 |
| Desire for exchange between the physicians | 5 |
| Missing vaccination certificate | 3 |
| Satisfaction with the vaccination process | 13 |
| Satisfaction with the consultation | 25 |
| Desire for advice | 63 |
| Request for information material | 18 |
| Specialist-specific wishes - More knowledge about the disease | 44 |
| Preference for vaccination | 43 |
| Changing attitudes due to the pandemic | 1 |
| Own attitude | 46 |
| Attitude of the treating physicians | 46 |
| Wishes for the future | 0 |
| Wishes for future treatment | 20 |
| General wishes for future vaccinations | 48 |
